# Subcortical life, evolution of flattened body, and constrained mating posture in the earwig *Platylabia major* (Insecta: Dermaptera: “Anisolabididae”)

**DOI:** 10.1371/journal.pone.0293701

**Published:** 2023-11-02

**Authors:** Yoshitaka Kamimura, Chow-Yang Lee

**Affiliations:** 1 Department of Biology, Keio University, Yokohama, Japan; 2 Urban Entomology Laboratory, Vector Control Research Unit, School of Biological Sciences, Universiti Sains Malaysia, Gelugor, Penang, Malaysia; University of Illinois Chicago Department of Biological Sciences, UNITED STATES

## Abstract

Many animals take advantage of the shaded, humid, and protected environments in subcortical spaces, i.e., thin spaces under the loosened bark of dead trees. Permanent inhabitants of subcortical spaces often show specialized morphologies, such as a miniaturized or dorsoventrally flattened body. However, the evolutionary consequences of these specialized morphologies on behavioral, ecological, and life-history traits have been little studied. We studied the mating biology and anatomy of *Platylabia major* (usually placed in the family Anisolabididae), which is an obligate inhabitant of subcortical spaces with a paper-like flattened body, and compared them with those of two thicker, spongiphorid earwigs, *Nesogaster amoenus* and *Paralabellula curvicauda*. Mating trials in various settings showed that *Pl*. *major* requires thin spaces sandwiched by two planes to accomplish genital coupling and insemination. In contrast, the thicker species, although also frequently found in subcortical spaces, could mate on a single horizontal plane due to the ability of the male to twist its abdomen through approximately 180°. Examination by micro-computed tomography and a reagent-based clearing technique revealed no substantive differences in the configuration of mid-abdominal musculature between the species. The dorsal and lateral muscles of *Pl*. *major*, which are almost parallel to the antero-posterior body axis for accommodation within the thin abdomen, seemed incapable of producing the power to twist the abdomen. The abdominal musculature conforms to a simple pattern in both male and female earwigs, which is repeated in each of the pregenital segments. We conclude that small differences in the range of motion of each abdominal segment can result in large differences in possible mating postures and positions. Surgical experiments also demonstrated that both right and left penises of *Pl*. *major* are competent and used for insemination with no lateral bias, as in most other earwigs with twin penises studied to date.

## Introduction

Many animals take advantage of the shaded, humid, and protected environments under the loosened bark of dead trees (hereafter referred to as subcortical spaces), either temporarily or throughout the entire life course [1–7]. Inhabitants of thin subcortical spaces often show specialized morphologies, such as a miniaturized or dorsoventrally flattened body [1, 2, 4, 8, 9]. These modifications in body plans can cause coevolutionary changes in other behavioral, ecological, and life-history traits. For example, among tropical skink species (Reptilia: Squamata: Scincidae: Lygosominae), dorsoventrally flat species that use rocky rather than subcortical environments compensate for the significant reduction of the abdominal volume by being “more full of eggs,” resulting in a similar clutch size to robustly built species [10]. In contrast, Pelegrin et al. [11] reported that flattened *Tropidurus* reptile species, which are also adapted to rocky habitats, show a narrower range of prey items and lower reproductive output than robustly built congeners. However, given animal diversity, many other examples, processes, and morphological modifications could be studied. Addressing these would help refine our understanding of the coevolutionary rinks between behavioral traits and specialized morphologies.

Many insect species of the order Dermaptera, commonly known as earwigs, also use subcortical spaces. For example, based on material collected in extensive field surveys in 2012–2013, our previous studies [12, 13] reported 32 earwig species from Penang Island (299 km^2^), Peninsular Malaysia, in tropical Asia. Among these, we collected at least one sample of 20 species (62.5%), representing five families, from subcortical spaces of dead logs (Table 1). Multiple families of earwigs include species with highly flattened bodies, indicating parallel evolution (we expand on this in the “Discussion”).

**Table 1:** Dermapteran species, of which at least one sample was collected from subcortical spaces of dead logs in Penang Island, Peninsular Malaysia, with their familial and subfamilial classifications. The data are based on unpublished details of the records reported by Kamimura et al. (2016a, b) [12, 13].

Species	Family (Subfamily)
*Diplatys annandalei* Burr, 1911	Diplatyidae (Diplatyinae)
*Cranopygia pallidipennis* (de Haan, 1842)	Pygidicranidae (Pygidicraninae)
*Echinosoma denticulatum* Hincks, 1959	Pygidicranidae (Echinosomatinae)
*Echinosoma sumatranum* (de Haan, 1842)	Pygidicranidae (Echinosomatinae)
*Echinosoma roseiventre* Kamimura & Nishikawa, 2016	Pygidicranidae (Echinosomatinae)
*Gonolabis electa* Burr, 1910	Anisolabididae (Anisolabidinae)
*Metisolabis punctata* (Dubrony, 1879)	Anisolabididae (Brachylabidinae)
*Platylabia major* Dohrn, 1867	"Anisolabididae" (Platylabiinae)*
*Allostethus indicum* (Burmeister, 1838)	"Labiduridae" (Allostethinae)**
*Chaetospania anderssoni* Brindle, 1971	Spongiphoridae (Labiinae)
*Chaetospania javana* Borelli, 1926	Spongiphoridae (Labiinae)
*Chaetospania huisiangi* Kamimura & Nishikawa, 2016	Spongiphoridae (Labiinae)
*Chaetospania thoracica* (Dohrn, 1867)	Spongiphoridae (Labiinae)
*Paralabellula boettcheri* (Borelli, 1923)	Spongiphoridae (Labiinae)
*Paralabellula curvicauda* (Motschulsky, 1863)	Spongiphoridae (Labiinae)
*Paralabellula rotundifrons* (Hincks, 1954)	Spongiphoridae (Labiinae)
*Spirolabia pilicornis* (Motschulsky, 1863)	Spongiphoridae (Labiinae)
*Nesogaster amoenus* (Stål, 1855)	Spongiphoridae (Nesogastrinae)
*Spongovostox mucronatus* (Stål, 1860)	Spongiphoridae (Spongiphorinae)
*Spongovostox semiflavus* (Bormans, 1894)	Spongiphoridae (Spongiphorinae)

*See the main text for a discussion of the phylogenetic placement and the subfamilial name.

**See Haas & Kukalová-Peck (2001) [60] and Kamimura & Lee (2014b) [52] for discussions about the phylogenetic placements of the genus *Allostethus*.

Members of the genus *Sparatta* Audinet-Serville, 1839 (Spongiphoridae) possess a markedly flattened body [14]. Briceño and Eberhard [15] examined courtship and mating behaviors of 13 Neotropical earwig species from four families and reported the courtship and copulation of *Sparatta bolivari* de Bormans, 1880: “After rubbing, the male often attempted to turn upside down. This was not possible in our glass-topped observation chambers, but in a narrow tunnel with one glass side, a courting male succeeded in turning upside down, and he immediately moved backward to place the ventral surface of his cerci against the ventral side of the female’s abdomen and copulate.” However, they observed only one instance of copulation in this species, and it is unclear whether a tunnel-like environment is indispensable for its reproduction. Both males and females of the family Apachyidae also have a remarkably dorsoventrally flattened body [16]. Shimizu and Machida [17] observed the mating of *Apachyus chartaceus* (de Haan, 1842) in a cylindrical plastic vessel containing a cylinder made of nonwoven fabric to simulate their subcortical habitats in the wild. They reported that *A*. *chartaceus* mated in an end-to-end manner, with the male and female being dorsoventrally reversed, and that they did not mate or lay eggs in vessels lacking the fabric cylinder.

However, not all earwig species that inhabit subcortical spaces have a flattened body, indicating a spectrum in the extent of adaptation to the environment. We studied the mating biology and morphology of the earwig species *Platylabia major* Dohrn, 1867 (Anisolabididae), almost obligately found in subcortical spaces and characterized by markedly flattened body. To understand the evolutionary consequences of body flattening, we examined copulation and insemination success of *Pl*. *major* in various settings, including thin spaces sandwiched by two woody planes that mimic subcortical spaces. For comparison, we also examined the mating behavior of two earwig species of similar size, *Nesogaster amoenus* (Stål, 1855) (Spongiphoridae) and *Paralabellula curvicauda* (Motschulsky, 1863) (Spongiphoridae). Although these latter two species have less flattened bodies, they are frequently found together with *Pl*. *major* under the bark of dead logs in rubber plantations in Peninsular Malaysia. To elucidate the possible causes of the observed behavioral differences among these species, we examined the external and internal morphology, particularly the abdominal musculature, using micro-computed tomography (μCT), scanning electron microscopy (SEM), and a reagent-based clearing technique. Furthermore, male *Pl*. *major* possesses laterally paired penises (right penis and left penis), while only a single penis is located at the center of genitalia in the other two studied species [14, 16, 18–21]. We conducted an experiment with surgical ablation of one penis from male *Pl*. *major* to test the functional competence of the laterally paired penises. Based on the results, we also discuss insights into the evolution of this unique genus.

## Materials and methods

### Ethical note

The experiments described below, including those with dissection and surgical procedures, were conducted following general ethical guidelines for invertebrates [22, 23]. Based on the results of previous similar research cases [24–28], the number of insects subjected to experimental procedures, including surgical operations, was minimized to levels that could withstand statistical analysis. Additionally, appropriate anesthesia (treatment with ice-cold water) and euthanasia (by freezing) procedures were carried out during surgery and dissection. This study was conducted with the approval of the Economic Planning Unit, Malaysia (Reference No. UPE: 40/200/19/2844).

### Study populations and husbandry

The following studies were conducted mainly using a laboratory stock population of *Pl*. *major*, derived from a single female collected on Penang Island, Malaysia. After initial growth, the stock population was divided and maintained in two separate plastic vessels (100 mm in diameter, 100 mm in height) with plaster of Paris at the base and several pieces of tree bark as harborage under laboratory conditions of 25°C ± 1°C with a 12-h light–dark cycle. They were provided with water and commercial cat food *ad libitum*. Large-sized nymphs were routinely separated into a smaller plastic vessel (80 mm in diameter, 40 mm in height, with plaster of Paris at the base but without harborage) and maintained under the same laboratory conditions. They were checked for imaginal eclosion every 2 days. Newly emerged adults were housed according to sex and maintained as described above.

Similarly, study populations were established from a single female of *Pa*. *curvicauda* (Langkawi Island, Malaysia) and *N*. *amoenus* (Penang Island, Malaysia). The body of *Pa*. *curvicauda* vary in color (blackish brown or reddish chestnut) (e.g., Burr 1911). These two morphs may represent different species (Y.K., unpublished data), and the latter morph was used in the present study. The adult females collected with the abovementioned method were confirmed to have no sperm in the spermatheca (*Pl*. *major*, *N* = 28; *Pa*. *curvicauda*, *N* = 22; *N*. *amoenus*, *N* = 23).

### External and internal morphology

A total of six specimens (*N* = 1 each sex of each species) were used for μCT examination. The specimens preserved in 70% ethanol were dehydrated by passage through an ascending series of 80–99.5% ethanol for > 24 h, soaked in *t*-butanol three times for > 1 h each time, and sublimation dried (JFD-320; JEOL Ltd., Tokyo, Japan). Scans of these specimens were made over 180° rotation with images every 0.2° under a current of 200 μA and a source voltage of 50 kV (SkyScan1272 scanner; Bruker, Kontich, Belgium). Three frames, taken with no filter, were averaged. The resultant images had voxel sizes of 2.0–4.0 μm. The resulting X-ray projections were reconstructed using NRecon (Bruker) and visualized using CTVox (Bruker). Similarly treated samples were also examined by SEM (JSM-6510; JEOL) at an acceleration voltage of 15 kV after coating with gold by ion sputtering (Hitachi E101; Hitachi Ltd., Tokyo, Japan).

As thin sclerotized structures, such as the phragmas of sclerites to which many muscles are inserted, are difficult to observe by μCT, a reagent-based clearing technique was also used to observe the abdominal musculature (*N* ≥ 3 for each sex of each species). Briefly, specimens (not in copula) were dehydrated and soaked in BABB solution (benzyl alcohol/benzyl benzoate = 1:2) at 25°C for more than 6 months following the method of Kamimura and Mitsumoto [29] but without embedding in an agarose block. The specimens were then observed under a differential interference contrast (DIC) microscope (BX53, 100–400×; Olympus, Tokyo, Japan) to determine their detailed abdominal musculature.

### Mating behavior and insemination success

**Fig 1:**
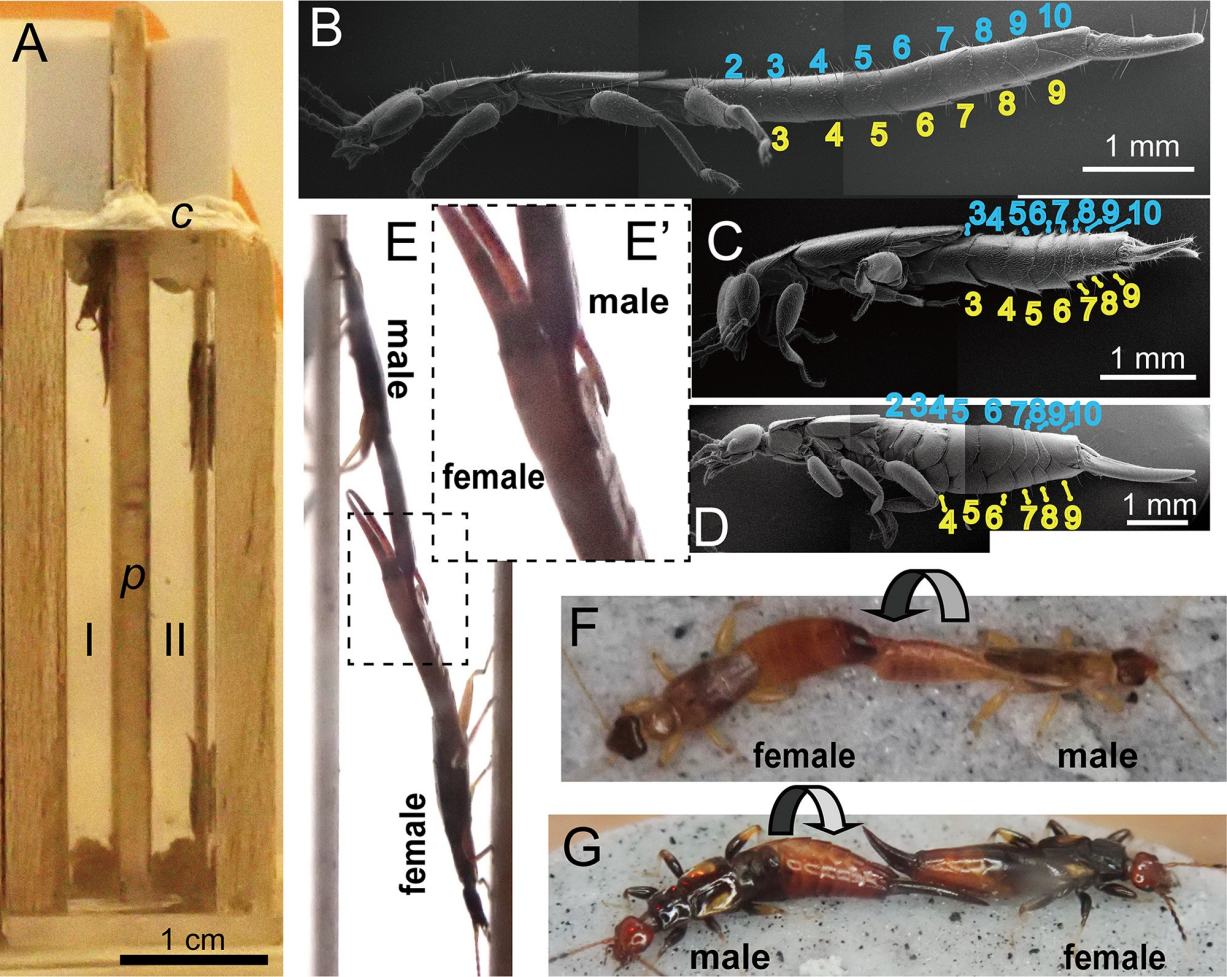
Experimental setting for the observation of mating in thin spaces mimicking under-bark environments (**A**), male habitus (**B**–**D**) and mating postures (**E**–**G**) of *Platylabia major* (**B**, **E**, **E’** [enlarged view of **E**]), *Paralabellula curvicauda* (**C**, **F**), and *Nesogaster amoenus* (**D**, **G**). Two thin spaces (I and II; each 10 mm in length, 4 mm in width, and 45 mm in height) separated by a balsawood partition (*p*) and sealed with a block of plastic clay (*c*; TA-380, Kokuyo, Osaka, Japan), were observed simultaneously from the lateral side through the clear acrylic resin wall. In **B**-**D**, the ordinal numbers of abdominal segments are indicated for tergites (light blue) and sternites (yellow). The arrows in **F** and **G** indicate that the male rotates the abdomen in a clockwise direction (viewed from the head of the male) to establish genital contact.

Mating behavior and insemination success of *Pl*. *major* were observed for pairs consisting of a virgin female and virgin male under three settings: **(a)** on a horizontal flat plane without harborage (16 pairs); **(b)** on a vertical flat plane without harborage (15 pairs); or **(c)** in a thin space sandwiched by two vertical flat planes (13 pairs). In **(a)** and **(b)**, the base of plaster of Paris in a plastic container (30 mm in length × 20 mm in width × 10 mm in height) was thoroughly covered with a thin balsawood board (1 mm thick). For setting details of **(c)**, see Fig 1A. Pairing commenced at 18:15 and ended at 18:15 the next day. Thirty minutes before the start of pairing, females were released into a mating arena for acclimation, whereas the males were introduced immediately before the experiments. For setting **(c)**, the behavior of pairs was recorded from the lateral side using a video camera (GZ-MG980S or GZ-R300; Victor, Kanagawa, Japan) with a time-lapse recording function (one frame per 2 s). Filming during dark periods (20:00–08:00) was conducted under dim red light. Based on the results of preliminary observations and equipment limitations, video recording in the settings **(a)** and **(b)** was not conducted.

Similar video recording was also conducted for virgin pairs of *Pa*. *curvicauda* (10 pairs) and *N*. *amoenus* (11 pairs), but on a flat base of plaster of Paris, placed horizontally in a small cylindrical plastic vessel (35 mm in diameter, 7 mm in height). As a pilot study showed that pairs of *N*. *amoenus* mate highly frequently, a 1-h video recording (in light periods: 13:00–17:00) was conducted for this species. To confirm that *Pl*. *major* cannot copulate at all on a single plane regardless of the base material (plaster of Paris or balsawood), an additional experiment was also performed in which 11 virgin males and 15 females were released *en masse* into a single arena (73 mm in diameter, 35 mm in height; base made of plaster of Paris) for 15 days.

After pairing, all the females were sacrificed by placing them in a freezer (−20°C). The spermatheca was removed from the female abdomen, mounted on a glass slide with a drop of phosphate-buffered saline, and checked for the presence of detectable sperm under the DIC microscope.

Males of the two thicker species, *Pa*. *curvicauda* and *N*. *amoenus*, twisted the abdomen almost 180° to bring the genitals on the ventral side into contact with those of the female mate (see Results). The direction of abdominal twisting, either clockwise (CW) or counterclockwise (CCW), viewed from the head of the male (Fig 1F and 1G), was also recorded for these species at the initiation of copulation. To examine whether each male individual consistently twisted the abdomen in the CW or CCW direction, the absolute value of the laterality index (ABLI: [28, 30, 31]) was calculated for each male according to the following formula:

$$ABLI=|LI|=\left|\frac{Q_{CW}-Q_{CCW}}{Q_{CW}+Q_{CCW}}\right|$$ (Eq 1)

where *Q*_CW_ and *Q*_CCW_ are the number of occasions where the focal male twisted the abdomen in the CW and CCW direction, respectively. This index can vary from 0 (no lateralization when *Q*_CW_ = *Q*_CCW_) to 1 (complete lateralization when *Q*_CW_ = 0 or *Q*_CCW_ = 0) (see “Statistical analysis” for the statistical test procedures for this index).

### Laterality and competency of paired penises

Male *Pl*. *major* possesses a pair of penises, contrary to the other species studied (two spongiphorids with a single penis). When in repose, either the right or left penis points posteriorly and is therefore ready to establish the end-to-end copulation position, while the other bends at the base to point anteriorly [18, 21, 32, 33]. Penis laterality (i.e., genital configuration as right-penis ready [Rr] or left-penis ready [Lr]) was examined under a stereomicroscope (SZX16; Olympus). To confirm the generality of the observed penis laterality, the offspring of four wild-caught females (ISB1–4; Penang Island, Malaysia) were examined in addition to the main study population.

A surgical experiment was conducted using virgin adults from the stock population to examine whether only one of the two penises is functionally competent. Ninety males (3–30 days old) were randomly assigned to one of three surgical treatment groups, i.e., ablation of the right or left penis or sham operation (control), before examination of penis laterality (Rr or Lr). The left or right penis and interior virga were removed from a male with a pair of fine forceps under anesthesia with ice-cold water, effectively forcing the male to be right-handed (Rh) or left-handed (Lh), respectively. The genitalia of control (Ctl) males were extracted from the genital chamber, but neither penis was ablated. Accordingly, six groups were prepared: Lr-Ctl, Lr-Lh, Lr-Rh, Rr-Ctl, Rr-Lh, and Rr-Rh.

After a recovery period of 5–6 days, each male was paired with a virgin female (7–35 days old) for 7 days in a separate rearing vessel (80 mm in diameter, 40 mm in height) with a base of plaster of Paris and three pieces of dried tree bark to provide thin spaces. The specimens were euthanized by placing them in a freezer (−20°C). They were then dissected under a stereomicroscope to check the penis status (Rr or Lr) and possible damage to the genitalia due to surgery (males) or to examine the presence of sperm in the spermatheca (females). We amputated the right penis from 15 Rr and 15 Lr males and the left penis from 15 Rr and 15 Lr males, together with 14 Rr-Ctl and 16 Lr-Ctl males (*N* = 90 in total). A total of 29 trials were excluded from the analyses because of cannibalism, death of the male or female specimen during the recovery or pairing period, dissection failure, or detection of unintended damage in the male genitalia (S1 Table).

### Statistical analysis

All statistical analyses, except for testing ABLI, were conducted using R version 4.0.2 [34]. A binomial test was used for each population or brood to test for deviations from the null hypothesis that the ratio of Rr to Lr males was 1:1. Significance thresholds in multiple comparisons were corrected using the false discovery rate (FDR) [35]. Fisher’s exact probability test was used to compare ratios between groups.

To analyze the effects of surgical treatments on male insemination success, generalized linear models were fitted using the “glm()” function by incorporating the type of surgical treatment (Ctr, Rh, or Lh), the penis state before surgical treatment (Rr or Lr), and the interaction term as possible explanatory factors. We adopted a binomial error structure and a logistic link function. Insemination status in females was recorded as “0” (no detectable sperm in the spermatheca) or “1” (sperm detected in the spermatheca) as the response variable. We adopted a backward variable-selection strategy, starting with the full model, including all the explanatory variables. The residual deviance of the full model was 37.485 with 55 degrees of freedom, with no significant indication of overdispersion (*P* = 0.904, *NS*; tested using the “testDispersion()” function in the DHARMa package; [36]). The significance of the interaction term was tested using the likelihood ratio test (LRT) between the full model and the model from which the interaction term was removed. Then, the main effect factors were sequentially removed in a stepwise manner using the LRT. The same analysis was applied for *post hoc* pairwise multiple comparisons between treatment groups when a significant treatment effect was detected. Significance thresholds in multiple comparisons were corrected using the FDR.

We tested the deviation of an average value of ABLI from a series of null hypotheses of random binomial expectations using a Monte Carlo simulation method (20,000 replicates) using scripts written in Python 3.8.3 (see [28] for details).

## Results

### Mating and insemination on two-dimensional planes or in sandwiched spaces

**Fig 2:**
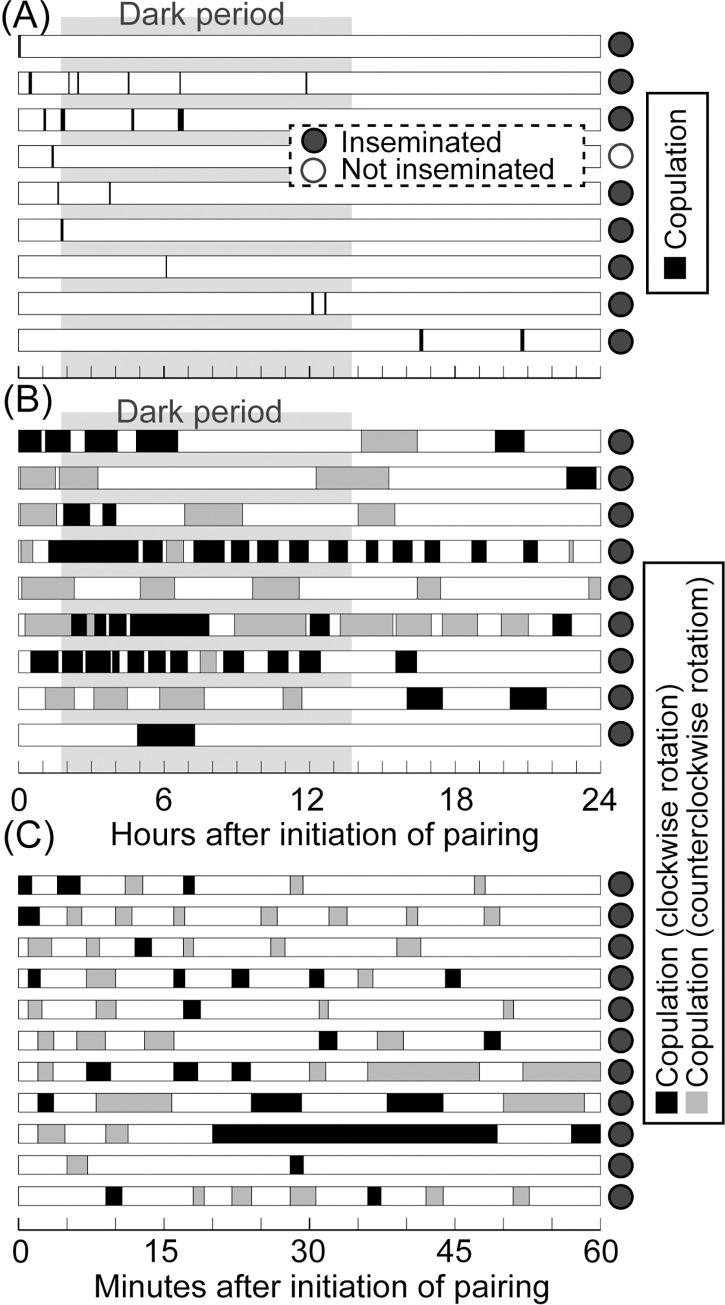
Copulation duration and frequencies of *Platylabia major* (**A**: nine pairs), *Paralabellula curvicauda* (**B**: nine pairs), and *Nesogaster amoenus* (**C**: 11 pairs). Each rectangle represents the time window of video recording for each pair, in which the filled parts indicate copulation bout. Filled and open circles at the right side of each bar indicate that the female was inseminated or uninseminated after the observation period, respectively. Dark periods are indicated by gray shading (in **A** and **B**). The copulation bouts shown in black and gray in **B** and **C** indicate the direction of male abdominal rotation (clockwise or counterclockwise, respectively) recorded at the initiation of each copulation. See the main text for details.

In 13 male/female pairs of *Pl*. *major* filmed in the setting mimicking subcortical spaces, nine pairs mated one to six times (mean ± standard deviation [SD] = 2.2 ± 1.7 times) during the 24-h observation period. As in many other earwig species [15, 20], all males of this species actively courted the female by directing their cerci (forceps) to those of the female. However, no copulation occurred in the other four pairs. The duration of a single copulation varied extensively, with a mean ± SD of 274.3 ± 181.7 s (range, 90–770 s, *N* = 20), yielding a total mating duration of 609.4 ± 602.4 s over the 24-h observation period (range, 95–1940 s, *N* = 9: Fig 2A). Of the females that copulated at least once, a female with a single copulation of 235 s had no detectable sperm in the spermatheca, while the other eight, with a minimum cumulative copulation duration of 95 s, were successfully inseminated (Fig 2A).

As both male and female genitalia are located on the ventral side of the post-abdomen, male earwigs usually rotate their abdomen through approximately 180° to establish genital contact [15, 20]. However, in all 20 recorded matings, male *Pl*. *major* did not rotate the abdomen. Instead, the males on a vertical wall gently warped the abdomen dorsally to establish genital contact with a female perching on the opposite wall (Fig 1E and 1E).

In contrast to the moderately high insemination success rate in the thin space (eight of 13 pairs observed), all female *Pl*. *major* remained uninseminated when they paired with a male in the settings with only a single vertical or horizontal plane without harborage (Fisher’s exact probability test: *P* = 0.00058 [*P* = 0.00124 after adjustment for multiple comparisons]; *N* = 14 each after removal of one and two females with dissection failure, respectively). In addition, none of the 15 females housed with 11 males for 15 days on a base of plaster of Paris showed any detectable sperm in the spermatheca (Fisher’s exact probability test: *P* = 0.00041 [*P* = 0.00124 after adjustment for multiple comparisons]).

Males of two spongiphorid species, *N*. *amoenus*, and *Pa*. *curvicauda*, successfully established genital coupling on the smooth, horizontal floor of the mating arena without harborage, resulting in the insemination of all females except for one pair of *Pa*. *curvicauda* (Fig 2B and 2C; see Table 2 for mating statistics). They achieved this by twisting their abdomen approximately 180° in the CW or CCW direction (viewed from the head of the male) (Fig 1F and 1G). In total, male *N*. *amoenus* rotated the abdomen in the CW direction in approximately half of the copulations (24 of 63 occasions). There was also no significant individual laterality (ABLI = 0.322; *P* = 0.766; Monte-Carlo simulation with 20,000 replicates), indicating that each male twisted the abdomen in the CW or CCW direction with equal frequency. However, in *Pa*. *curvicauda*, although CW and CCW abdominal rotations were almost equally frequent (40 and 26 occasions, respectively), males showed significant bias toward a specific direction (ABLI = 0.517 [calculated and tested by omitting one pair that mated only once]; *P* = 0.033).

**Table 2:** Number and duration of copulation observed in virgin male/female pairs of *Platylabia major*, *Paralabellula curvicauda*, and *Nesogaster amoenus*.

Species	*Platylabia major*	*Paralabellula curvicauda*	*Nesogaster amoenus*
Duration of filming	24 hours	24 hours	1 hour
No. of pairs filmed	13	10	11
No. of pairs with no copulation	4	1	0
No. of copulations observed	2.2 ± 1.7 (range, 1–6; *N* = 9)	7.3 ± 4.6 (range, 1–15; *N* = 9)	5.7 ± 1.7 (range, 2–8; *N* = 11)
No. of females inseminated	9	9	11
Duration of single copulation bout	274.3 ± 181.7 s (range, 90–770 s, *N* = 20)	74.7 ± 46.3 min (range, 10–222 min, *N* = 66)	168.2 ± 235.0 s (range, 56–1761 s, *N* = 63)
Cumulative duration of copulation bouts	609.4 ± 602.4 s (range, 95–1940 s, *N* = 9)	547.4 ± 282.0 min (range, 142–1145 min, *N* = 9)	963.5 ± 647.7 s (range, 210–2246 s, *N* = 11)

### Laterality of paired penises and their competence in sperm transfer

**Fig 3:**
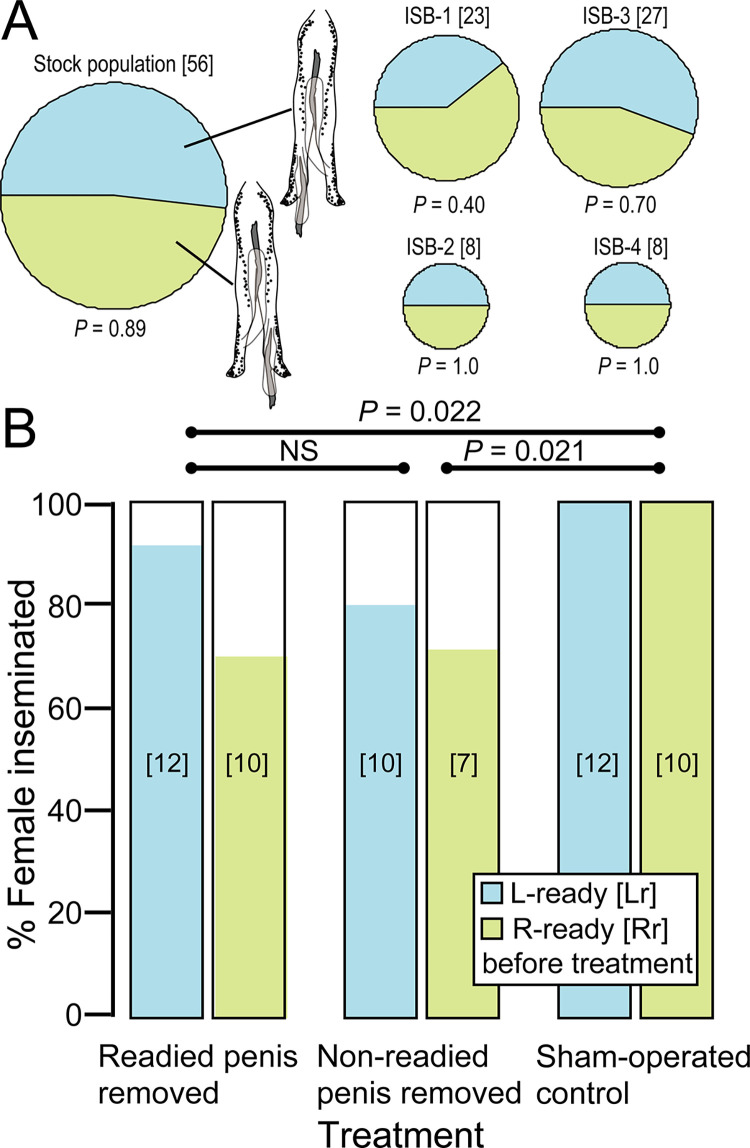
(**A**) Penial laterality of four F1 generation broods and a stock population of *Platylabia major* derived from Penang Island, Malaysia. Light green and light blue parts represent right-ready (Rr) and left-ready (Lr) penis status, respectively. The size of each circle represents the number of males examined (shown in brackets). The *P*-values shown are those for deviation from Rr:Lr = 1:1, all of which were > 0.05 even without correction of significance thresholds for multiple comparisons. **(B)** Effects of artificial penis‐ablation treatments of *Pl*. *major* on insemination success. The light-green and light-blue bars show the results for males that were Rr or Lr before the surgical treatment, respectively. Sample sizes are shown in brackets. The *P*-values shown are those after adjustment of thresholds for multiple comparisons.

We examined the laterality of the paired penises, one of which is considered ready for copulation by pointing posteriorly, for five groups of *Pl*. *major*. None of the stock population or four broods (F_1_) from possibly unrelated females of *Pl*. *major* showed significant bias in penis laterality from the null hypothesis of Rr (right-ready): Lr (left-ready) = 1:1 when significance thresholds were adjusted appropriately for multiple comparisons (Fig 3A). Overall, 50.0% (*N* = 122 in total) were Rr.

We conducted a surgical experiment to confirm that both paired penises are functionally competent in *Pl*. *major*. Overall, 79.5% of males (31 of 39) from which one of the paired penises had been ablated successfully inseminated a virgin female over a 7-day cohabitation period, while 100% insemination success was recorded for the 22 sham-operated males. Although the insemination success rate varied significantly among treatments (ablation of readied penis *vs*. ablation of unreadied penis *vs*. sham operation; LRT: *Χ*^2^_2_ = 8.0, *P* = 0.018), it was unaffected by the penis status (Rr or Lr) before the surgical treatment (*Χ*^2^_1_ = 1.5, *P* = 0.22) or by the interaction of both factors (*Χ*^2^_2_ = 0.42, *P* = 0.81). Following adequate adjustment of significance thresholds for multiple comparisons, the insemination success rate was not significantly different between the cases of ablation of the readied and unreadied penis, but both were lower than that of the sham-operated males (Fig 3B). At the end of the experiment, the penis that had not been removed was ready for use in all 39 surgically treated males, indicating that flipping of penial direction occurred only in the cases where a readied penis had been ablated in this experiment (*N* = 22).

### Interspecific comparisons of abdominal morphology and musculature

To infer the causes of the observed differences in mating behaviors among the three earwig species, we observed the external and internal morphologies using SEM and μCT, respectively. Due to the telescopic nature of the dermapteran abdomen, accurate measurement of its dimensions is challenging [37, 38]. However, the approximate aspect ratio (length of abdomen excluding forceps divided by its height) in male *Pl*. *major* has an extremely high value of approximately 9.0, in contrast to 4.4 and 2.9 for male *Pa*. *curvicauda* and *N*. *amoenus*, respectively (Fig 1B–1D). The abdominal tergites and sternites in *Pa*. *curvicauda* and *N*. *amoenus* are arranged alternately with the *N*th tergite preceding the corresponding sternite (Fig 1C and 1D). In contrast, the *N*th sternite of the mid-abdomen is located almost directly under the *N*th tergite in *Pl*. *major* (less-alternating arrangement) (Fig 1B).

**Fig 4:**
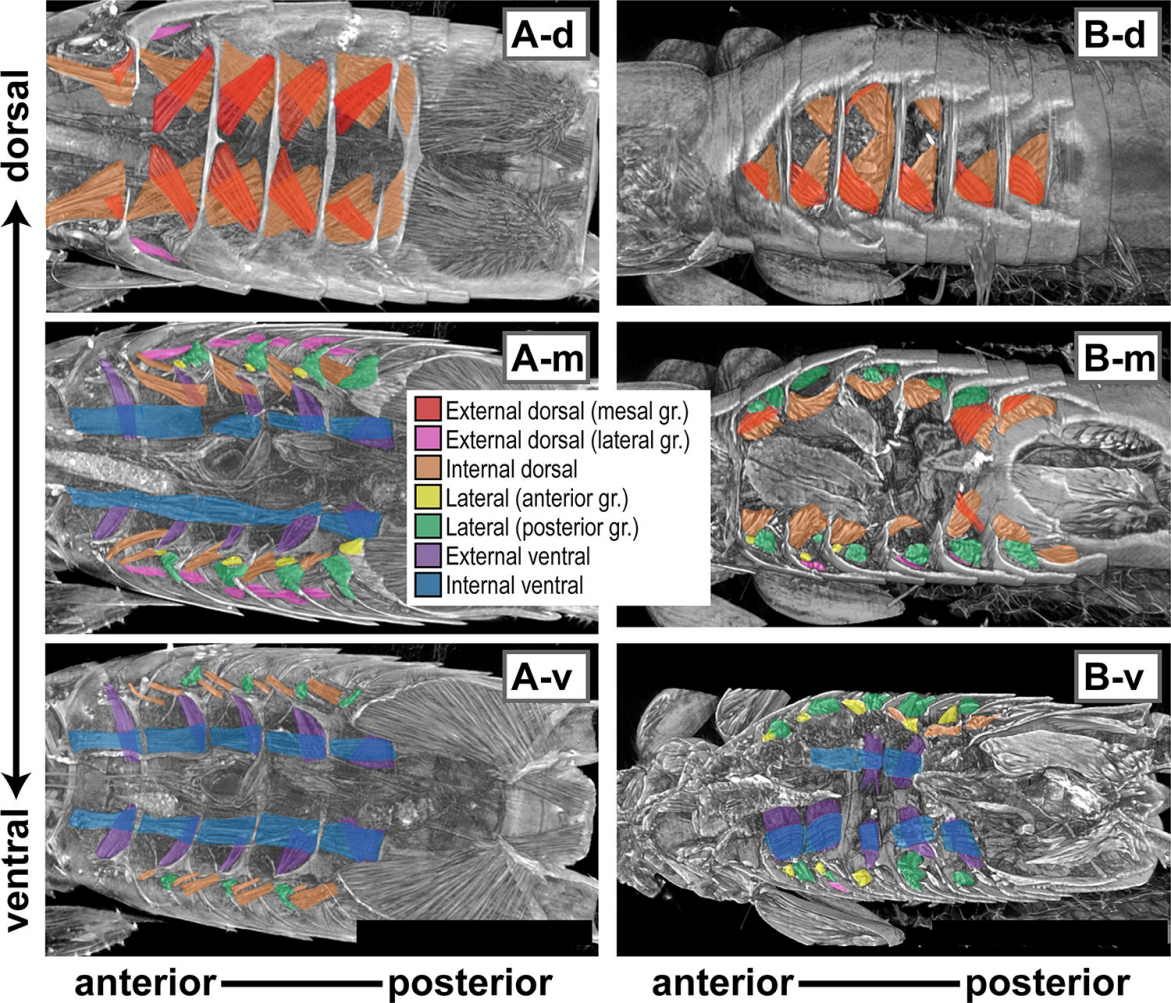
The abdominal structures observed and reconstructed by micro-computed tomography for male *Platylabia major* (A-d, A-m, A-v) and male *Nesogaster amoenus* (B-d, B-m, B-v). Major muscle bundles possibly responsible for abdominal movements were divided into seven subgroups and highlighted in different colors. See Table 1 and the main text for further details.

Regardless of the substantive interspecific differences in the shape of the abdomen, we found that the abdominal musculature is similar among these three species. As in previous studies ([39–43]; see Discussion for details), we recognized six major categories of muscles in the mid-abdominal pregenital segments (III–VII in females; III–VIII in males) (Table 3), highlighted in different colors in Fig 4.

**Table 3:** List of major muscles detected in each abdominal segment of the examined earwigs, with a summary table of previous studies on the dermapteran abdominal musculature*.

Position: Relation to segments	Designated color in Figs 4 and 5	This study	Popham (1959) [43]**	Ford (1923) [39]***	Klug & Klass (2007) [42]****
Dorsal (Tergal): Intersegmental	Red + Pink	External dorsal muscle	Dorsal oblique muscle	Outer tergal muscle	#11, #12
Dorsal (Tergal): Intersegmental	Orange	Internal dorsal muscle	Lateral oblique muscle	Inner tergal muscle	#10
Ventral (Sternal): Intersegmental	Blue	Internal ventral muscle	Median ventral muscle	Inner sternal muscle	#7
Ventral (Sternal): Intersegmental	Purple	External ventral muscle	Oblique ventral muscle	Outer sternal muscle	#8
Lateral (tergo-sternal): Intrasegmental	Yellow	Anterior lateral muscle	Dorso-lateral muscle	Tergo-sternal muscle (tertiary + quarternary)	#2, #3
Lateral (tergo-sternal): Intrasegmental	Green	Posterior lateral muscle	Tergo-sternal muscle	Tergo-sternal muscle (primary + secondary)	#1, #16

* Maki [40] also studied the thoracic muscles and those of the anterior abdominal segments (segments II–VI) of *Euborellia annulipes* (Lucas, 1847) (= *Anisolabis annulipes* in Maki’s study: Anisolabididae) and *Pa*. *curvicauda* (= *Labia curvicauda* in Maki’s study), although the anteriormost abdominal segments showed some modifications related to their degenerative trends in Dermaptera.

**Study species: *Forficula auricularia* Linnaeus, 1758 (Forficulidae)

***Study species: *Forficula auricularia* (Forficulidae)

**** Study species: *Hemimerus vosseleri* Rehn & Rehn, 1935 (Hemimeridae), a viviparous species that lives phoretically on the giant pouched rat (*Cricetomys*), with reference to unpublished observation of free-living *Labidura riparia* (Pallas, 1773) (Labiduridae)

**Fig 5:**
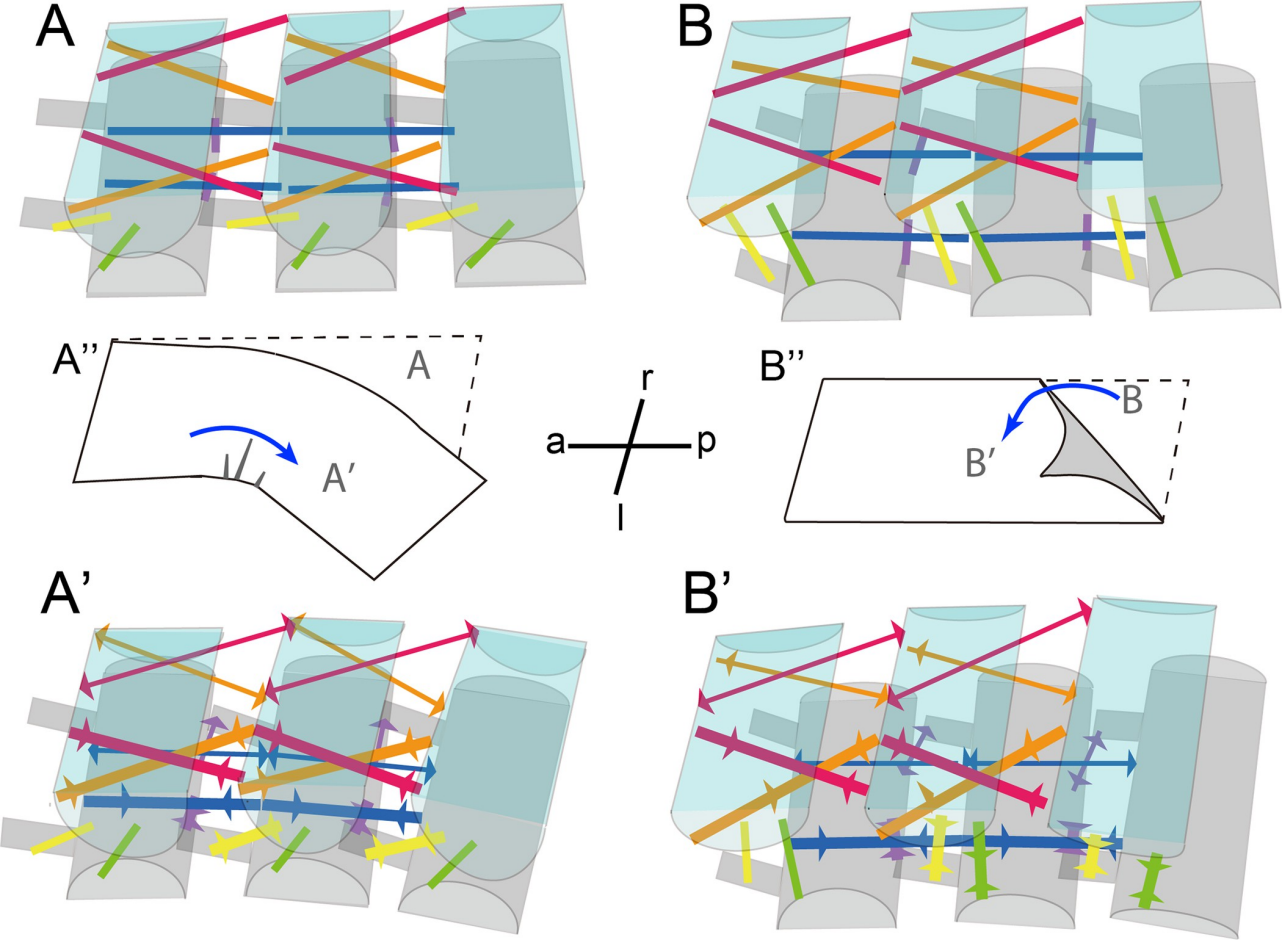
Schematics of horizontal abdominal bending in *Platylabia major* (**A** to **A’**) and twisting of mid-abdominal segments in thick-bodied dermapteran species (**B** to **B’**). The major muscle bundles are shown in different colors, as in Fig 4. The muscle bundles that are expected to be contracted or stretched to accomplish the respective abdominal movements are shown by thicker lines with converging arrowheads or thinner lines with diverging arrowheads, respectively, in **A’** and **B’**. Note that the shapes and sizes of the tergites (light blue) and sternites (gray) of mid- abdominal segments are simplified and not shown to scale. Paper-like models of horizontal bending (**A”**) twisting (**B”**) are also shown with indications of the anterior (a)–posterior (p) and right (r)–left (l) body axes.

Except for the alary muscles for pulsation (not shown in Figs 4 and 5), two groups of dorsal muscles connect the successive abdominal terga [39, 42]. Most dorsally, a large group of external dorsal muscles (red in Fig 4; mesal group) originate from near central anterior parts of a tergite and are inserted near the lateral ends of the phragma of the next tergite. Another group of external dorsal muscles runs laterally (pink in Fig 4; lateral group) at distinct angles from the corresponding mesal group. Internal to these, internal dorsal muscles (orange in Fig 4) run from each tergite lateral end to near the center of the phragma of the next tergite. Accordingly, when observed from the dorsal side, this muscle makes “laterally paired crosses” with the mesal group of the external dorsal muscles in each abdominal segment (Fig 4A–4D and 4B–4D). Given the thick body, the internal dorsal muscles of *Pa*. *curvicauda* and *N*. *amoenus* run almost diagonally, from near the ventrolateral corner of the *N*th tergite to the dorsal-center of *N+1*th tergite (Fig 4B-4M). Klug and Klass [42] noted that this muscle could also be divided into at least two groups with distinct angles from each other (see Fig 4A-4M) and that this may be correlated with the anterolateral bending of the abdomen characteristic of Dermaptera in the lower Neoptera.

Only intersegmental muscle bundles have been reported for Dermaptera as the ventral coxosterno-coxosternal muscles [42]. A large pair of longitudinal muscle bundles run almost parallel to the anteroposterior body axis (hereafter a–p axis) on each side of the ventral nerve cord (internal ventral muscles: blue in Fig 4). A pair of apophyses (or apodemes; hereafter sternal apophyses) protrude from the anterior end of each abdominal sternite. This structure is obliquely connected to the previous sternite by a sheet of external ventral muscles (purple in Fig 4).

In the present study, we detected only intrasegmental lateral muscles connecting the *N*th tergite and *N*th sternite (Figs 4 and 5), similar to other free-living earwigs studied to date ([39, 41–43]; but see Klass’s [41] study for exceptions in *H*. *vosseleri*). These lateral muscles can be divided into two categories with different innervation patterns [42], both of which consist of at least two types of muscles [39, 41, 42]. The anterior group inserts into the outer side of the sternal apophyses (yellow in Fig 4). The posterior group (green in Fig 4) is usually longer and stouter than the anterior group, connecting the tergite and sternite (near the widest parts) almost perpendicular to the a–p axis (Fig 4). Given the less-alternating arrangement of the abdominal tergites and sternites (Fig 1B) and the longer but less-raised anterior apophyses of the sternites, both groups of lateral muscles are notably inclined in *Pl*. *major*, running almost parallel to the dorsal muscles and internal ventral muscles (Fig 4A-4M).

## Discussion

### Evolutionary adaptations for life under the bark

With the whole body markedly flattened dorsoventrally, the life of *Pl*. *major* under bark seems almost two-dimensional (2D). However, our mating experiments under different settings showed that their life cycles could not be completed on a single 2D plane: genital coupling of *Pl*. *major* was observed only in thin spaces sandwiched by two planes, although the lower mating frequency compared to the other two species (Fig 2) suggests that the observation setting (4 mm in height) was suboptimal for this species. Although not quantitative, previous studies on *S*. *bolivari* (Spongiphoridae) and *A*. *chartaceus* (Apachyidae) indicated that these species, which are characterized by a highly flattened body, also require a thin space for reproduction [15, 17]. Previous authors have placed the genus *Platylabia*, which comprises the monotypic subfamily Platylabiinae (see [44–46] for deliberation on the subfamilial name), in the family Anisolabididae (= Carcinophoridae) [16, 19, 33, 44, 47–51]. However, Kamimura et al. [12] found well-developed 8th-segment gonapophyses and a pair of structures possibly representing the 9th-segment laterocoxa in the female genitalia of *Pl*. *major*, suggesting its affinity to the family Labiduridae (see also [52]). Although there have been no detailed studies on the phylogenetic position of this group in the Dermaptera, Kamimura et al. [53] concluded that this genus is not very close to Anisolabidinae (Anisolabididae). There is no evidence that these three genera (*Sparatta*, *Apachyus*, and *Platylabia*) form a monophyletic clade, and the requirement of habitats consisting of two adjacent planes has likely evolved multiple times in Dermaptera, along with the evolution of the markedly flattened bodies. Flattened bodies for subcortical life are considered to have evolved multiple times from typically shaped generalists in the beetles of Histeroidea [8], indicating that body shape is less informative for estimating the phylogenies of these insects.

Interestingly, although frequently found with *Pl*. *major* in the same subcortical space in Malaysian forests, two other species of earwigs examined in this study do not require two planes for copulation: the males of *Pa*. *curvicauda* and *N*. *amoenus* can twist their abdomen to bring the genitalia on the ventral side into contact with the female genitals. Although they have a much thicker body (i.e., much lower aspect ratio), these species are likely able to use subcortical spaces because of their smaller size (Fig 1B–1D). Alternatively, they may use wider parts of subcortical spaces than *Pl*. *major*. As earwigs inhabiting subcortical spaces show a wide variety of body shapes and sizes, we need further studies to investigate possible segregation in their microhabitats when multiple species coexist.

Concerted coevolution of morphological and behavioral traits between the sexes is likely indispensable for the evolutionary changes in mating positions. A key question is whether the end-to-end mating position with the male inverted (Fig 1E) represents a novel evolutionary trait unique to species with a highly flattened body. End-to-end positions with abdominal twisting by the male are also known for Diptera, Heteroptera, strophandrous Hymenoptera, and Lepidoptera [54, 55]. This type of mating, which is common in Dermaptera, is considered to be derived directly from the plesiomorphic “female-above” mating position or via “end-to-end with male inverted” [54, 55]. Interestingly, the latter mating position has also been observed sporadically for some earwig species that can mate on a single horizontal plane by male abdominal twisting. For example, Knabke and Grigarick [56] reported that male *Euborellia cincticollis* (Gerstaecker, 1883) turned over, rather than twisting the abdomen, for mating with a female only “if a support was available.” Therefore, the evolutionary development of the “end-to-end with male inverted” mating position would not have required any special behavioral modification. Instead, *Platylabia* and other flattened earwigs have lost the ability to mate in end-to-end positions with abdominal twisting.

### Why does *Pl*. *major* require two planes?

Regardless of the marked differences in their mating positions and requirements for two adjoining planes, our examination revealed essentially the same constitution of the abdominal musculature among the three species that show a large interspecific variation in the aspect ratio (= thinness: 9.0 in *Pl*. *major* to 2.9 in *N*. *amoenus*). There were also no differences between the sexes, except for some modifications associated with degeneration of the 8th and 9th abdominal segments in female adults of Dermaptera [57, 58]. As Klug and Klass [42] reviewed, there are only a few preceding studies on the abdominal musculature of Dermaptera (Table 3: [39, 41, 43]). Aside from some discrepancies among them, the major muscles in the mid-abdominal pregenital segments (III–VII in females; III–VIII in males) of free-living dermapterans can be divided into six categories (Table 3). Our observations confirmed a similar musculature for the three species studied here.

However, the marked differences in shapes of the abdominal tergites and sternites, together with their arrangements, can cause differences in possible mating postures and positions. First, nearly circular abdominal cross-section of *N*. *amoenus* and *Pa*. *curvicauda* seems to provide a larger range of motion of the *N+1*th segment when *N*th segment is fixed. Second, these morphological differences in the exoskeleton resulted in differentiation of the relative length and locations of the abdominal muscles between *Pl*. *major* and more thickly built species (*Pa*. *curvicauda* and *N*. *amoenus*).

For twisting of the abdomen, unilateral contraction of laterally paired, intersegmental muscles must cause downward movement of the *N+1*th segment in the contracted side (= upward movement of the opposite side) relative to the *N*th segment. For this, the muscles should run diagonally to the orientation of the abdominal segment series. If muscles are almost parallel to the plane containing the a–p and right-left (r–l) axes, unilateral contraction of the muscles can generate power only to swing the abdomen parallel to the plane. Accordingly, the internal dorsal muscles (highlighted in orange in Figs 4 and 5) seem crucial for the observed differences in mating postures. These muscles are well-developed and run diagonally in each lateral half of the abdominal segment in *Pa*. *curvicauda* and *N*. *amoenus*. However, in *Pl*. *major*, the corresponding muscles lie almost in a plane parallel to the a–p and r–l axes.

The left (right) side of the body serves as the axis of rotation when the male twists the abdomen in the CW (CCW) direction (Fig 1F and 1G). Unilateral contraction of lateral muscles, which run perpendicular to the a–p axis, may aid abdominal twisting in the thicker species. The abdominal tergites and sternites in *Pa*. *curvicauda* and *N*. *amoenus* are arranged alternately, with the *N*th tergite preceding the *N*th sternite. Therefore, although all the lateral muscles are intrasegmental, their unilateral contraction can cause compaction of the lateral side that serves as the axis of rotation. In contrast, the corresponding muscles of *Pl*. *major*, which are largely inclined and therefore more parallel to the a–p axis, may reinforce the work of the dorsal and ventral muscles to swing the abdomen horizontally.

In summary, this study revealed no marked changes in the abdominal musculature of *Pl*. *major* in response to the evolution of their highly flattened body. Nevertheless, the abdominal musculature conforms to a simple pattern in both male and female dermapterans, which is repeated in each of the pregenital segments (Fig 4; [39, 41–43]). Thus, small differences in the range of motion of each abdominal segment can result in large differences in possible mating postures and positions.

### Insights from penis use patterns

The present study also revealed that males ready for use the right penis and those ready for use the left penis are equally frequent in multiple broods of *Pl*. *major*. In addition, although the surgically treated males from which one penis had been removed showed a lower insemination success rate than the sham-operated controls, males from which the readied penis was removed inseminated females as frequently as those from which the un-readied penis was removed. Therefore, the observed reduction in insemination success may have been attributable to presently unspecified detrimental effects of the surgical treatment. Both of the paired penises of *Pl*. *major* are likely competent for transferring sperm, as in five other earwig species with twin penises studied to date (*Diplatys flavicollis* Shiraki, 1908 [24]; *Echinosoma sumatranum* (de Haan, 1842) [26]; *Euborellia pallipes* Shiraki, 1906 [59]; [the name *E*. *plebeja* (Dohrn, 1863) was used in the paper, but see [53]]; *Labidura riparia* (Pallas, 1773) [25, 27]; and *Nala lividipes* (Dufour, 1828) [28]). Among them, *L*. *riparia* (Labiduridae) is known to be right-handed, i.e., males predominantly use the right penis for transferring sperm to the female spermatheca during copulation [25, 27], whereas males of the other species studied use the right and left penis equally frequently at the population level.

The present study showed that the characteristic mating posture of *Pl*. *major* without male abdominal twisting is not accompanied by population-level lateralization of penis use. In *L*. *riparia*, a characteristic membrane tightly connects the dextrally coiled base of the spermatheca and the dorsal wall of the vaginal region [27]. Although its evolutionary origin and functional significance for females are unclear, this unique structure is likely to have caused the right-handedness of penis use in *L*. *riparia* [27]. The base of the spermathecal duct is not fixed to other female structures with such a flange-like membrane in *Pl*. *major* [12], indicating that it can accept a right or left penis equally easily.

Each male of *N*. *lividipes* consistently used one of the paired penises, either the right or the left, showing strong lateralization at the individual level [28]. In future studies, this aspect should be examined by repeated inspection of the penis status of *Pl*. *major* and other earwig species with twin penises.

## Supporting information

S1 Table Complete data sets of the penis-ablation experiment, summarized in Fig 3B of the manuscript. (PDF)
