# Supplementary material for: Subcortical life, evolution of flattened body, and constrained mating posture in the earwig Platylabia major (Insecta: Dermaptera: “Anisolabididae”)
Source: PLoS One. 2023 Nov 2;18(11):e0293701. doi: 10.1371/journal.pone.0293701 (PMC10621853; doi:10.1371/journal.pone.0293701)
Supplement: S1 Table — (PDF) [file pone.0293701.s001.pdf]

S1 Table. Complete data sets of the penis-ablation experiment, summarized in Fig. 3B of the manuscript.

Male_code	Readied_penis_at_operation (Right [R] or Left [L])	Penis_Ablated (Right [R] or Left [L] os Sham [C])	Experiment_Type	Experiment_Type2	Treatment_success (1: Success; 0: Failure)	Insemination (1: With sperm; 0: No sperm)	Days_between_Operation_Cohabitation	Operation_success
1_1	L	R	L_R	Ab_Non_Ready	0 (Male genitalia damaged)	0	5	0
1_2	R	L	R_L	Ab_Non_Ready	1	0	5	1
1_3	R	C	R_C	Cont	1	1	5	1
1_4	L	R	L_R	Ab_Non_Ready	0 (Male genitalia damaged)	0	5	0
1_5	R	R	R_R	Ab_Ready	1	1	5	1
1_6	L	L	L_L	Ab_Ready	1	1	5	1
1_7	R	L	R_L	Ab_Non_Ready	0 (Male died)	NA	NA	NA
1_8	R	R	R_R	Ab_Ready	1	0	5	1
1_9	R	C	R_C	Cont	0 (Male died)	NA	NA	NA
1_10	R	L	R_L	Ab_Non_Ready	0 (Male genitalia damaged)	0	5	0
2_1	R	L	R_L	Ab_Non_Ready	0 (Male died)	NA	NA	NA
2_2	L	L	L_L	Ab_Ready	1	1	5	1
2_3	R	R	R_R	Ab_Ready	1	1	5	1
2_4	R	L	R_L	Ab_Non_Ready	0 (Female died)	NA	NA	NA
2_5	L	R	L_R	Ab_Non_Ready	1	1	5	1
2_6	R	C	R_C	Cont	1	1	5	1
2_7	R	L	R_L	Ab_Non_Ready	1	0	5	1
2_8	R	R	R_R	Ab_Ready	1	1	5	1
3_1	L	L	L_L	Ab_Ready	1	1	5	1
3_2	L	C	L_C	Cont	1	1	5	1
3_3	L	R	L_R	Ab_Non_Ready	0 (Male genitalia damaged)	0	5	0
3_4	L	R	L_R	Ab_Non_Ready	1	0	5	1
3_5	R	L	R_L	Ab_Non_Ready	1	1	5	1
3_6	R	L	R_L	Ab_Non_Ready	0 (Male genitalia damaged)	0	5	0
3_7	L	C	L_C	Cont	1	1	5	1
3_8	L	L	L_L	Ab_Ready	1	1	5	1
4_1	L	R	L_R	Ab_Non_Ready	0 (Male genitalia damaged)	0	5	0
4_2	R	L	R_L	Ab_Non_Ready	0 (Male died)	NA	NA	NA
4_3	L	R	L_R	Ab_Non_Ready	1	1	5	1
4_4	L	C	L_C	Cont	1	1	5	1
4_5	R	C	R_C	Cont	1	1	5	1
4_6	L	L	L_L	Ab_Ready	1	1	5	1
5_1	L	R	L_R	Ab_Non_Ready	1	1	5	1
5_2	R	L	R_L	Ab_Non_Ready	1	1	5	1
5_3	L	L	L_L	Ab_Ready	1	1	5	1
5_4	L	C	L_C	Cont	1	1	5	1
5_5	L	R	L_R	Ab_Non_Ready	0 (Male genitalia damaged)	0	5	0
5_6	R	C	R_C	Cont	1	1	5	1
5_7	L	R	L_R	Ab_Non_Ready	1	1	5	1
5_8	R	R	R_R	Ab_Ready	0 (Male genitalia damaged)	0	5	0
6_1	R	R	R_R	Ab_Ready	1	0	5	1
6_2	L	C	L_C	Cont	1	1	5	1
6_3	R	C	R_C	Cont	1	1	5	1
6_4	L	L	L_L	Ab_Ready	1	1	5	1
6_5	R	C	R_C	Cont	1	1	5	1
7_1	L	C	L_C	Cont	1	1	5	1
7_2	L	L	L_L	Ab_Ready	1	1	5	1
7_3	R	R	R_R	Ab_Ready	1	0	5	1
7_4	L	L	L_L	Ab_Ready	1	1	5	1
7_5	R	L	R_L	Ab_Non_Ready	0 (Male died)	NA	NA	NA
7_6	R	R	R_R	Ab_Ready	0 (Male died)	NA	NA	NA
7_7	L	L	L_L	Ab_Ready	1	0	5	1
7_8	R	C	R_C	Cont	0 (Female dissection failed)	NA	NA	NA
8_1	R	C	R_C	Cont	1	1	6	1
8_2	L	C	L_C	Cont	1	1	6	1
8_3	L	R	L_R	Ab_Non_Ready	1	1	6	1
8_4	R	C	R_C	Cont	0 (Female dissection failed)	NA	NA	NA
8_5	R	L	R_L	Ab_Non_Ready	1	1	6	1
8_6	R	C	R_C	Cont	1	1	6	1
8_7	R	R	R_R	Ab_Ready	1	1	6	1
8_8	R	R	R_R	Ab_Ready	0 (Male died)	NA	NA	NA
10_1	L	C	L_C	Cont	0 (Male died)	NA	NA	NA
10_2	L	L	L_L	Ab_Ready	0 (Male died)	NA	NA	NA
10_3	L	C	L_C	Cont	1	1	5	1
10_4	L	C	L_C	Cont	1	1	5	1
11_1	R	C	R_C	Cont	0 (Female died)	NA	NA	NA
11_2	L	R	L_R	Ab_Non_Ready	1	0	5	1
11_3	L	R	L_R	Ab_Non_Ready	1	1	5	1
11_4	R	L	R_L	Ab_Non_Ready	1	1	5	1
12_1	L	L	L_L	Ab_Ready	0 (Female died)	NA	NA	NA
12_2	L	L	L_L	Ab_Ready	1	1	5	1
12_3	L	C	L_C	Cont	1	1	5	1
12_4	L	L	L_L	Ab_Ready	1	1	5	1
12_5	R	R	R_R	Ab_Ready	1	1	5	1
12_6	L	R	L_R	Ab_Non_Ready	1	1	5	1
12_7	R	L	R_L	Ab_Non_Ready	0 (Male died)	NA	NA	NA
12_8	L	C	L_C	Cont	0 (Male died)	NA	NA	NA
12_9	R	R	R_R	Ab_Ready	0 (Male genitalia damaged)	0	5	0
12_10	L	C	L_C	Cont	0 (Male genitalia malformed)	NA	NA	NA
12_11	R	L	R_L	Ab_Non_Ready	1	1	5	1
12_12	L	C	L_C	Cont	0 (Female genitalia malformed)	NA	NA	NA
12_13	R	C	R_C	Cont	1	1	5	1
12_14	R	R	R_R	Ab_Ready	1	1	5	1
13_1	L	C	L_C	Cont	1	1	5	1
13_2	R	R	R_R	Ab_Ready	1	1	5	1
13_3	L	L	L_L	Ab_Ready	0 (Male died)	NA	NA	NA
13_4	R	C	R_C	Cont	1	1	5	1
13_5	R	R	R_R	Ab_Ready	0 (Male died)	NA	NA	NA
13_6	L	C	L_C	Cont	1	1	5	1
13_7	L	R	L_R	Ab_Non_Ready	1	1	5	1
